# Maternal well-being and family adaptation during COVID-19 in fragile X syndrome

**DOI:** 10.3389/fpsyt.2022.952118

**Published:** 2022-08-23

**Authors:** Heather Fielding-Gebhardt, Rebecca Swinburne Romine, Shelley Bredin-Oja, Nancy Brady, Steven F. Warren

**Affiliations:** ^1^Schiefelbusch Institute for Life Span Studies, University of Kansas, Lawrence, KS, United States; ^2^Department of Communication Sciences and Disorders, Minot State University, Minot, ND, United States; ^3^Department of Speech-Language-Hearing: Sciences and Disorders, University of Kansas, Lawrence, KS, United States

**Keywords:** anxiety, COVID-19, *FMR1* premutation, fragile X syndrome, adaptation

## Abstract

Mothers of children with fragile X syndrome are at increased risk of experiencing anxiety and depression due to potential genetic risk and to stress associated with parenting a child with significant behavioral, emotional, and educational support needs. During the initial shutdown and subsequent restrictions of the COVID-19 pandemic, mothers of children with fragile X reported experiencing elevated levels of anxiety and depression relative to their usual levels of well-being. Many indicated that the negative consequences of exposure to COVID-19 and related stressors, as well as the impacts of the pandemic on their family, directly affected their anxiety and depression. Mothers reported on specific sources of distress as well as potential sources of resilience and positive adaptation that occurred during the first year of the COVID-19 pandemic.

## Introduction

This study examined the impacts of the COVID-19 pandemic on the mental health of mothers of children with fragile X syndrome. Fragile X syndrome is caused by an elongated CGG trinucleotide repeat on the *FMR1* gene, located on the X chromosome. Individuals with between 55 and 200 CGG repeats carry the premutation and individuals with >200 repeats have the full mutation (1, 2). Here, we focus on mothers of children with the full mutation (FXS), who are themselves carriers of the premutation or the full mutation. Current prevalence estimates suggest that as many as 1 in 151–208 women in the United States carry the premutation (3, 4).

Women with the *FMR1* premutation are at elevated risk for neuropsychiatric disorders, including anxiety and depression (5–9), and the stress of raising a child with FXS can exacerbate these symptoms (10–13). Specifically, mothers of children with FXS experience elevated rates of anxiety, depression, and affective disorders relative to mothers of children without disability (14, 15). They may also report more pessimism and concerns with the future, as well as increased family conflict, relative to mothers of children with Down syndrome (16). As such, mothers who carry the *FMR1* premutation represent a group which may benefit from mental health services, especially during stressful or difficult periods.

The COVID-19 pandemic reached the United States in March 2020, causing communities across the nation to enact restrictions on public interaction, including school closures and restrictions of therapeutic services. Families of children with developmental disabilities were greatly impacted by these restrictions. Specifically, 52.3% of families of individuals with intellectual and developmental disabilities reported losing access to speech therapy, 57.2% to occupational therapy, 62.9% to ABA therapy, 73.6% to social skills services, and 89% to other recreation services (17). This immense loss in therapeutic services may have negatively impacted the mental health of families of children with neurodevelopmental disorders such as FXS.

Pandemic-related changes in social support and supplementary services may have compounded challenges in caring for a child with a disability that were already present pre-pandemic. During COVID-19, caregivers of children with intellectual disability reported experiencing significantly greater levels of anxiety and depression than caregivers of typically developing children, with over 40% of the former group endorsing moderate to severe levels of anxiety and depression compared to ∼ 10% of the latter (18). Additionally, anxiety and depression were negatively associated with measures of social support, household income, and house size (proxy for family size), and were positively associated with stress (caregiver, financial, and lockdown stress) (18). As such, the additional stress added by pandemic restrictions and loss of social support and services likely negatively impacted mental health in parents/caregivers of children with developmental disabilities.

Specific to families with FXS, one study reported that children with FXS experienced worsening sleep quality and increased behavioral problems during the first 5 weeks of the Italian full lockdown (19). Families also reported reduced access to external support and services. However, mothers did not report changes in their self-efficacy as parents. Although this study provided important information on how the COVID-19 pandemic has affected families with FXS, the authors did not directly probe mental well-being in mothers of children with FXS, who are at known increased risk of mental health problems. Furthermore, despite the stability in parental self-efficacy, there is a need to understand the specific challenges that parents and families of children with FXS have experienced during the COVID-19 pandemic.

Because mothers of children with FXS are at increased genetic risk of experiencing anxiety and depression, and because occurrence of anxiety and depression can be exacerbated by parenting stress and demands, the purpose of this study was two-fold: to characterize mental well-being of *FMR1* premutation mothers during the COVID-19 pandemic and identify potential sources of risk and resilience toward pandemic-related changes in mental health. This set of analyses utilizes a unique cohort of mothers of children with FXS who have been part of an ongoing longitudinal study of parenting and child development in FXS. As such, we considered current (i.e., pandemic) levels of anxiety and depression as well as changes relative to past levels, which enabled us to identify changes in mental health symptomology that is related to the COVID-19 pandemic and describe potential sources of risk and resilience for each family.

## Methods

### Participants

Thirty-six mothers of children with FXS provided data for these analyses. Their ages ranged from 41 to 59 years of age, with a mean age of 50.22. Two mothers had FXS, two had mosaicism for the pre- and full mutations, and the remaining 32 had the premutation. Among mothers with the premutation, the CGG repeat length ranged from 74 to 130, as confirmed through blood sample analyses. Because there were relatively few mothers with full mutation alleles, we did not consider maternal responses by genetic groups. Mothers were predominantly white and non-Hispanic (89%) and 61% had household incomes greater than $79,999. One mother was white and Hispanic, two were Black and non-Hispanic, and one was Black, Hispanic, and Pacific Islander. Children (8 girls) ranged in age from 16.6 years to 20.75 years of age, with a mean age of 19. Fifteen of the children had FXS and autism co-morbidity, as measured by the CARS and ADOS-2 [see Fielding-Gebhardt et al. (20) for more details].

Study procedures were approved by the University of Kansas Human Research Protection Program, which ensures all legal and ethical standards necessary to protect the rights, well-being, and privacy of research participants, and in accordance with IRB standards, participants provided informed consent prior to participating in research activities.

### Procedure

The participants were part of an ongoing longitudinal study at the University of Kansas. In the parent study, mothers and their children with FXS were visited in their homes up to 8 times over a 17-year period, with one additional remote data collection conducted through the mail. Initial recruitment for the parent study took place through a parent listserv, a national registry, advertising at national conventions, and through parent support networks. Participants were initially recruited because they had a child with FXS.

For this study, data were collected remotely through the mail between October 2020 and January 2021. Pre-pandemic average levels of anxiety and depression were calculated from two data collection visits (roughly 2012 and 2018) and one remote assessment (2015). Six participating families did not complete the remote assessment. Otherwise, all participants provided data at all time points.

### Measures

#### Anxiety and depression

The Center for Epidemiological Studies – Depression scale [CES-D; Radloff (21)] was used to measure maternal symptoms of depression. This 20-item assessment asks mothers to indicate how frequently they have experienced symptoms of depression (e.g., feeling lonely, feeling sad, crying spells, etc.) over the past week. Items are scored on a scale from 0 to 3, with higher scores indicating more severe symptoms of depression. A clinical cut-off of 16 indicates that an individual with a score ≥16 may be at risk for clinical depression.

The original Profile of Mood States tension and anxiety subscale [POMS-TA; McNair et al. (22)] was used to measure maternal symptoms of tension and anxiety. There are 9 items on the tension and anxiety subscale which ask the mother to indicate how she was feeling during the past week. Items were scored from 0 to 4, with higher scores indicating more severe symptoms of anxiety. Nyenhuis et al. (23) reported a clinical cut-off of 17.2 (equal to 1.5 standard deviations above the standardization mean in a normative sample of adult women). Mothers with POMS-TA scores >17.2 were considered to have clinically significant symptoms of anxiety.

#### COVID-19 survey

The COVID-19 Exposure and Family Impact Scales [CEFIS; Center for Pediatric Traumatic Stress (24); Kazak et al. (25)] were used to measure families’ exposure to COVID-19 and their perceptions of its impact. Mothers completed the survey between October 2020 and January 2021, between 7 and 10 months following the national outbreak of the pandemic and the onset of nationwide COVID-19 restrictions. Mothers were instructed to consider their families’ experiences during the pandemic from March 2020 to present. For this survey, “families” referred to the mother, her child with FXS, those living in their household (i.e., father, siblings), extended family, and close friends who were considered like family. Because this measure was rapidly developed following the onset of the COVID-19 pandemic, at the time this manuscript was written there were no clinical cut-offs. However, normative data and psychometrics are provided in an article by Kazak et al. (25).

There are 25 CEFIS-Exposure items asking participants about exposure and related events such as stay at home orders, changes in income and/or employment, and whether family members contracted or had symptoms of COVID-19. Higher scores on the Exposure scale indicate higher exposure to potentially traumatic aspects of the pandemic. The CEFIS-Impact scale measures the perceived impact of the pandemic on the family. Ten CEFIS-Impact items are rated along a 4-point scale with higher scores indicating more negative impact. We included one additional item in the Impact scale, “How has the COVID-19 pandemic affected your emotional well-being, specifically depression?” In addition to the Exposure and Impact items, two CEFIS-Distress items are rated along a 10-point scale and assess the severity of COVID-19-related distress the mother and child each experienced. Again, higher scores indicate more distress. In addition to the CEFIS items, we asked mothers to elaborate on their answers to the Exposure and Impact scales. The final item on the CEFIS asks mothers to expand on their experiences during the COVID-19 pandemic and discuss other effects of COVID-19 not covered in the rest of the items.

The open-ended and elaboration questions were qualitatively coded using a conventional content analysis approach [Hsieh and Shannon (26)]. Two researchers (HF-G and SB-O) read all the responses and identified common themes in the qualitative answers. We then created a coding scheme (available upon request) based on these themes. Each researcher independently scored all participants’ answers, and then compared scores. When there were disagreements on codes, the two researchers agreed by consensus.

## Results

### COVID-19 pandemic effects and changes

Mothers reported substantial individual differences in exposure to and impact of COVID-19. The average level of exposure on the CEFIS-Exposure scale was 7.61, with a range from 2 to 17. Over three-quarters of mothers reported that they had stay at home orders, their child’s school was closed, their child’s education was disrupted, or that they missed an important family event such as a vacation or graduation, see Table 1. Over half of the families had a family member who worked outside the home in an essential personnel role. The next most commonly endorsed CEFIS-Exposure items were (1) a family member was exposed to a positive COVID-19 case, (2) family self-quarantined due to travel or exposure, (3) family was unable to care for or visit another family member, and (4) family income decreased. The total number of mothers who endorsed each CEFIS-Exposure item is presented in Table 1.

**TABLE 1:** CEFIS-Exposure scale items and frequencies.

Item	Description	# Reporting	% Reporting
1	Stay at home order	33	91.7
2	Schools/childcares closed	36	100
3	Education disrupted	32	88.9
4	Unable to visit/care for family member	16	44.4
5	Family lived separately	7	19.4
6	Someone moved into home	3	8.3
7	Had to move out of home	0	0
8	Family member worked outside home/essential worker	21	58.3
9	Family member in healthcare providing direct care	8	22.2
10	Difficulty getting food	2	5.6
11	Difficulty getting medicine	0	0
12	Difficulty getting healthcare	3	8.3
13	Difficulty getting other essentials	7	19.4
14	Self-quarantined due to travel or exposure	16	44.4
15	Family income decreased	11	30.6
16	Family member cut back work hours	9	25
17	Family member required to stop working	8	22.2
18	Family member permanently lost job	6	16.7
19	Family lost health insurance/benefits	1	2.8
20	Missed and important family event	27	75
21	Family member exposed to positive COVID-19 case	14	38.9
22	Family member had symptoms or COVID-19 diagnosis	7	19.4
23	Family member hospitalized for COVID-19	3	8.3
24	Family member in ICU for COVID-19	2	5.6
25	Family member died from COVID-19	2	5.6

**FIGURE 1:**
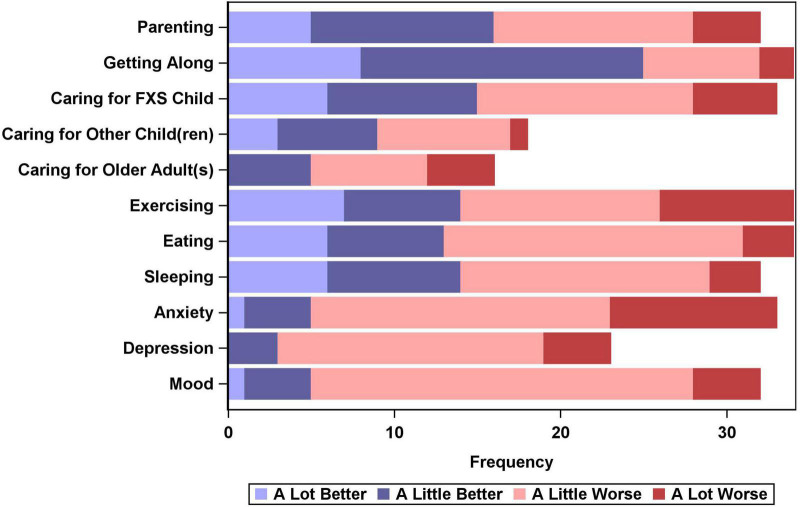
Distribution of CEFIS-impact scale item responses.

The CEFIS-Impact scale contained 11 items, 10 in the original version of the assessment along with one additional item (depression) that was added for this study. Each CEFIS-Impact item was rated along a 4-point scale (1 = a lot better, 2 = a little better, 3 = a little worse, and 4 = a lot worse). Mothers could also select “N/A” which we interpreted to mean “does not apply to me/my family” or “no change.” The distribution of scores is presented in Figure 1. While most mothers reported positive changes in “getting along,” many also reported negative impacts for most of the other items, including nearly 77% who reported worsening anxiety, 55% who reported worsening depression, and 74% who reported worsening mood. Anxiety, depression, and mood were all significantly more likely to be reported as “a little worse” or “a lot worse,” while sleeping, eating, and getting along were significantly more likely to have improved, with mothers more frequently reporting these activities got “a little better” or “a lot better.”

Mothers reported variable levels of distress associated with the COVID-19 pandemic both for themselves and for their children with FXS. Twenty mothers (55.6%) endorsed distress scores >5 (along a 10-point scale, with 10 indicating highest distress) for their own distress and 18 (50%) endorsed distress scores >5 for their child’s distress.

#### Anxiety and depression

We compared mothers’ levels of anxiety and depression over time, specifically examining the differences between three pre-pandemic occasions and the COVID-19 occasion. Using mixed effect models, which are better able to account for expected within person covariance than repeated measure ANOVAs, we found that there were significant differences between pre-pandemic and pandemic era anxiety and depression scores. Average anxiety score during COVID was 9.89 (range 1–27) which was significantly higher than average anxiety scores in October 2017 (average = 6.92, range = 0–20, *p* = 0.01) and July 2015 (average = 6.93, range = 0–20, *p* = 0.01), but not significantly different than anxiety in December 2010 (average = 7.92, range = 1–23, *p* = 0.08). Depression scores were highest during COVID (average = 11.75, range = 1–38) and were significantly higher than in October 2017 (average = 6.89, range = 0–28, *p* = 0.000), July 2015 (average = 8.43, range = 0–29, *p* = 0.01), and December 2010 (average = 8.89, range = 0–27, *p* = 0.03).

**FIGURE 2:**
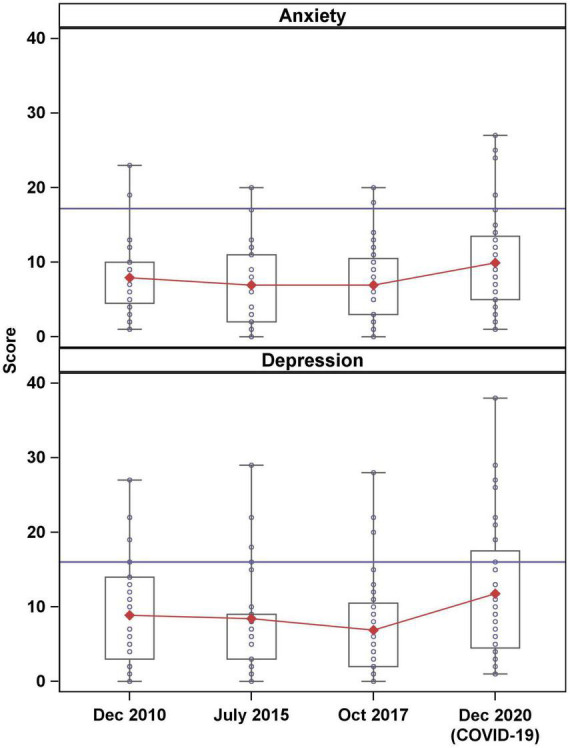
Distribution of anxiety and depression scores. Horizontal blue lines indicate clinical cut-offs and red lines connect mean scores over time.

In addition to elevated anxiety and depression scores during COVID, the distribution of scores increased, with more variability in the severity of symptoms. Figure 2 shows the distribution of mental health symptom scores prior to and during the pandemic. Three mothers (8.33%) met or exceeded the clinical cut-off on the POMS-TA prior to COVID-19, while six mothers (16.7%) had clinically significant symptoms of anxiety during the pandemic. Similarly, pre-pandemic, five mothers (13.89%) had clinically significant symptoms of depression on the CESD, but during the pandemic, ten mothers (27.8%) had clinically significant symptoms of depression.

### Sources of risk and resilience during the COVID-19 pandemic

Maternal anxiety was associated with COVID-19 Exposure (*r* = 0.41, *p* < 0.05), maternal Distress (*r* = 0.51, *p* < 0.01), and child Distress (*r* = 0.37, *p* < 0.05), such that mothers experienced more anxiety when Exposure and Distress were higher. This trend was the same for maternal depression, with mothers experiencing higher depressive symptoms when Exposure and Distress were higher (*r* = 0.46, *p* < 0.01, *r* = 0.54–0.62, *p* < 0.01). There was a small subset of mothers who indicated their anxiety (*n* = 5) and/or depression (*n* = 3) had improved. Mothers who had improvements in mental health did not differ from the rest of the sample in their likelihood of having had children with co-morbid autism, having sons, having lower income, having their child with FXS in an in-school versus online-only education setting, or in working either part- or full-time.

In addition to the mothers who reported reduced anxiety and/or depression, there was a subset of mothers who indicated that the overall impact of COVID-19 was positive. Seven mothers indicated that the overall impact of COVID-19 was slightly positive, noting improvements in exercise (*n* = 5), eating (*n* = 6), and sleeping (*n* = 7). None of them reported clinically significant levels of depression or anxiety, nor did they face increased economic anxiety due to COVID-19. None of these mothers was the parent of a child with co-morbid autism, and none reported that their offspring experienced an increase in problem behavior or had difficulty with the change in routine or schedule.

The CEFIS open-ended questions were examined for shared themes on mothers’ perceptions of the impact of the pandemic. Mothers’ answers fell along several themes within two categories: sources of stress/negative effects and positive adaptation. Many mothers reported on difficulties and negative experiences during the COVID-19 pandemic. Mothers often offered explanations for the negative effects, including distress due to lack of social interaction for their child(ren) (*n* = 21, 58%); increased child stress and decreased child mental well-being (*n* = 8, 22%); concerning child behaviors (*n* = 4, 11%) and difficulties surrounding a lack of routine or schedule (*n* = 11, 31%). Furthermore, mothers also cited being stuck at home as a source of distress. See Table 2 for examples of mothers’ reports from the open-ended questions. In contrast, many mothers reported that their child was able to positively adapt to the circumstances and that as a family they found positive experiences. Positive experiences during the pandemic period included increased family togetherness (*n* = 13, 36%), health improvements (*n* = 4, 11%), and child’s ability to adapt (*n* = 6, 17%).

**TABLE 2:** Examples of COVID-19 associated difficulties and positive adaptations reported by mothers.

Sources of stress/negative effects
**Reduction in access to services/activities** (*n* = 8, 20%)
[He] missed his community outing with his habilitation aide. The aide changed several times during COVID. It was hard for the agency to keep and find staff. [He] craved school and the social interaction.
Lack of involvement in social activities like special Olympics is greatly missed.
Being home [he] could not do any sports, which he loves to go to basketball, bowling, soccer, baseball practices and games. All activities were canceled for kids. Losing all the sports activities. [He] was not happy at all.
Libraries closed and are still closed.
**Social isolation** (*n* = 21, 58%)
No social interaction has been hard.
They are very isolated as we have been strictly quarantined since March, rarely seeing family or friends.
Lack of social interaction with friends is distressing.
[He] misses other people and engagement besides immediate family.
**Child well-being** (*n* = 8, 22%)
[He] regressed, became agitated and always nervous, hard to calm. Worried a lot and watched news religiously. Very high anxiety.
It bothered [her] a lot. Lots of anxiety.
My son’s anxiety and inability to enjoy activities has raised my anxiety.
In all my children, I saw and increase in anxiety and overall deterioration of mental health.
**Child behaviors and regression** (*n* = 4, 11%)
Behaviors resurfacing that haven’t been seen in years.
Regressed in personal care areas.
Lots of increased stimming and big decrease in social skills.
**Lack of routine** (*n* = 11, 31%)
Feels like constant disruptions to their routines and no community interaction.
Disruption in routine has been moderately difficult.
Every cancelation would bother [her]. She likes routine. She kept saying she’s going to “fight COVID and find a cure.”
**Family dynamics and conflict** (*n* = 6, 17%)
My [non-FXS] son couldn’t take virtual classes at home with a loud sibling- he had to go study in my mom’s basement.
The only other thing not mentioned is probably just everyone being home all the time together, we don’t get time to ourselves much as often. Mom and Dad are irritable, probably especially Mom. For instance, Dad is working from home and on a conference call right now and dog is barking at the squirrel outside. Mom is trying to get typical son out of bed for remote learning while trying to keep dog quiet.
Our house seemed smaller and smaller. Our 10- and 5-year-old with FX[S] seemed loud and stressful. It caused marriage stress. Had LOTS of trouble getting respite workers.
Being all together at home while trying to work or go to school has been difficult.
**Positive adaptation**
**Family togetherness** (*n* = 13, 36%)
It was great to get back to spending time together and eating all meals together.
With both of my children home we are all (parents included) communicating better. With little “hurry to this activity” between meals, etc., we are able to slow down and get clearer responses. Often thoughtful in a manner different than the past.
We did spend more quality time together – eating as a family, playing games.
On positive side- spent more time together as a family. We got to see online what my daughter is being taught. From speech, I learned how important PEC cards are – I created a schedule and menu of FX child’s favorite foods.
We’ve played a lot of games, slowed down, sent a lot of cards, and had a lot of long talks – all good. Both houses got puppies – also good.
The time together at home as a family was great b/c we unplugged and had more quality time.
It was great to get back to spending time together and eating meals all together
**Health improvements** (*n* = 4, 11%)
We all lost weight and exercise more because we have more time.
We’ve incorporated puzzles, daily reading, and exercise which has been great.
We sleep more, prepare better meals, spend more time together.
**Child adaptation** (*n* = 6, 17%)
He adapted very well to remote learning. He missed seeing his teachers/peers when school closed. Disappointed that other things changed but did pretty well adjusting.
My youngest son with FXS did online until school went in-person. He adapted beautifully and loves it all.
She is happy to be a quarantine champion because she doesn’t want to be in a COVID isolation sick.

## Discussion

The COVID-19 pandemic has had broad implications on mental health in general (27). Many mothers have taken on a higher burden of responsibility for childcare and have reported difficulty balancing work, childcare, and other family responsibilities, often to the detriment of their own mental health. Mothers in the general population have reported elevated anxiety and depression in the context of COVID-19 (28). Here, we demonstrate that mothers with the *FMR1* premutation had trouble adapting and coping during the pandemic, potentially resulting in decreases in mental well-being, but that many found sources of resilience and positive adaptation to the pandemic.

Although deterioration of mental health with age has been noted in *FMR1* premutation carriers (6, 9), our findings suggest that increases in anxiety and depression here were likely due to COVID-19 pandemic and associated stress, as our sample has demonstrated relative stability in mental health symptomology over time (11). The comparison between previous levels of anxiety and depression with COVID-19 pandemic levels, along with the number of mothers experiencing worsening anxiety and depression, suggest that the increase in symptoms of anxiety and depression is directly associated with the COVID-19 pandemic in our sample. Furthermore, mothers of children with neurodevelopmental disabilities such as FXS, may be at increased risk for mental health problems in general (9, 16, 29, 30), which could have been further aggravated during a global pandemic. Indeed, caregivers of children with intellectual disabilities experienced higher anxiety and depression than caregivers of children without intellectual disabilities during the COVID-19 pandemic (18). In one report, 44% of parents of children with special educational needs and disabilities reported feeling anxious during the pandemic, and a subset also reported feeling overwhelmed and fearful (31). Mothers in our sample were no exception, with many reporting elevated symptoms of anxiety and depression.

In addition to their own mental health concerns, mothers also reported concerns about their children’s well-being and behavior and about family dynamics, which is consistent with other studies on families of children with neurodevelopmental disabilities during COVID-19 (17, 18, 32). Specifically, families of children with autism have expressed concerns about their child being home all the time, about becoming sick, and about finances (32) – sentiments which were echoed in our families of children with FXS. Additionally, families of children with special educational needs and disabilities have reported stress due to caregiving demands, child behavior, changes in routine, and social isolation (31) – concerns which were again echoed in our sample.

One area of concern that has been highlighted during the COVID-19 pandemic is access to therapeutic, educational, and recreational services. Jeste et al. (17) reported a large drop in service availability during the early months of the pandemic, and Manning et al. (32) reported on the concerns of families of children with ASD, including school absence and worry about therapeutic services. All 36 families in our sample reported that schools or childcares closed in-person services, and nearly 90% reported that their child(ren) experienced disruption to their education. Many mothers also cited distress over reduced access to specific services and activities such as Special Olympics (Table 2).

Although we report on a unique cohort of mothers and children with FXS, there are several limitations to our study. Primarily, our sample is limited in racial, ethnic, and socioeconomic diversity. Given the racial health disparities seen in the United States and differences in access to care across communities, we cannot generalize our findings to all mothers of children with FXS. Our findings are further limited in scope as we did not compare maternal well-being and the impact of the COVID-19 pandemic in families of children with typical development. Additionally, data presented here were collected at the end of 2020, well before the Delta and Omicron waves began, so estimates of COVID-19 exposure and impact are likely to have changed since that time. However, our data do provide a compelling snapshot into the experiences of families with FXS at that time and highlight the support needs of all family members during difficult periods.

It is widely understood that the COVID-19 pandemic has had profound impact on the well-being of families, especially those of children with disabilities. Although many of our families experienced common negative consequences from the pandemic, many also reported positive adaptations. Mostly, mothers reported increased feelings of family togetherness, as they were able to take advantage of the shutdowns and spend more time with their children and partners, similar to other studies (31). Our families reported positive effects, such as the child’s ability to adapt to at-home learning and the family’s ability to slow down and improve their overall health. Thus, although the global pandemic has had incredible negative effects worldwide, there were still positives to be found in otherwise difficult situations. Our findings highlight the need to support families of children with developmental disabilities, specifically maternal mental health and access to services.

## Data availability statement

The raw data supporting the conclusions of this article will be made available by the authors, without undue reservation.

## Ethics statement

The studies involving human participants were reviewed and approved by the Human Research Protection Program at the University of Kansas. Written informed consent to participate in this study was provided by the participants’ legal guardian/next of kin.

## Author contributions

HF-G executed the study idea, collected, prepared, and analyzed the data, wrote the first draft of the manuscript, and incorporated comments and feedback from the co-authors. RSR prepared and analyzed the data and helped with the first and subsequent drafts of the manuscripts. SB-O collected and prepared the data and reviewed the manuscript. NB and SW collaborated in all stages and reviewed the manuscripts. All authors contributed to the article and approved the submitted version.
